# Unraveling the Origin of Magnetism in Mesoporous Cu-Doped SnO_2_ Magnetic Semiconductors

**DOI:** 10.3390/nano7110348

**Published:** 2017-10-25

**Authors:** Junpeng Fan, Enric Menéndez, Miguel Guerrero, Alberto Quintana, Eugen Weschke, Eva Pellicer, Jordi Sort

**Affiliations:** 1 Departament de Física, UniversitatAutònoma de Barcelona, E-08193 Cerdanyola del Vallès, Spain; junpeng.fan@gmail.com (J.F.); miguel.guerrero.hernandez@gmail.com (M.G.); alberto.quintana@uab.cat (A.Q.); eva.pellicer@uab.cat (E.P.); jordi.sort@uab.cat (J.S.); 2 Helmholtz-Zentrum Berlin für Materialien und Energie, Albert-Einstein-Straße 15, 12489 Berlin, Germany; eugen.weschke@helmholtz-berlin.de; 3 Institució Catalana de Recerca i Estudis Avançats (ICREA), Pg. Lluís Companys 23, 08010 Barcelona, Spain

**Keywords:** nanocasting, mesoporous SnO_2_ particles, diluted magnetic semiconductors

## Abstract

The origin of magnetism in wide-gap semiconductors doped with non-ferromagnetic 3d transition metals still remains intriguing. In this article, insights in the magnetic properties of ordered mesoporous Cu-doped SnO_2_ powders, prepared by hard-templating, have been unraveled. Whereas, both oxygen vacancies and Fe-based impurity phases could be a plausible explanation for the observed room temperature ferromagnetism, the low temperature magnetism is mainly and unambiguously arising from the nanoscale nature of the formed antiferromagnetic CuO, which results in a net magnetization that is reminiscent of ferromagnetic behavior. This is ascribed to uncompensated spins and shape-mediated spin canting effects. The reduced blocking temperature, which resides between 30 and 5 K, and traces of vertical shifts in the hysteresis loops confirm size effects in CuO. The mesoporous nature of the system with a large surface-to-volume ratio likely promotes the occurrence of uncompensated spins, spin canting, and spin frustration, offering new prospects in the use of magnetic semiconductors for energy-efficient spintronics.

## 1. Introduction

Diluted magnetic semiconductors (DMSs) have attracted an extraordinary technological and scientific interest since they may simultaneously exhibit ferromagnetism and semiconducting electric properties, hence being ideal candidates for novel applications in the field of spintronics [1,2,3]. However, the ferromagnetic behavior of some DMSs still remains rather intriguing [2]. Gaining insight into the magnetic properties of these materials may definitely facilitate their integration into spintronic and/or magnetoelectric devices [3]. Additionally, oxide DMSs are target materials to be magnetically manipulated using voltages [4]. In fact, voltage rather than current actuation (i.e., electric control of magnetism) might contribute towards the implementation of a new generation of energy-efficient spintronic technology, which could be of huge economic impact since energy losses by Joule effect would be minimized [5]. 

Among the numerous kinds of DMSs, current research focuses on: (i) narrow-gap III-V semiconductors, mostly GaAs and InAs, doped with Mn [2,6], and (ii) wide-gap oxides and nitrides doped with 3d transition metals either ferromagnetic (e.g., Fe, Co, or Ni) or non-magnetic, such as Cu [7,8]. While ferromagnetism in (i) is accepted to be carrier-mediated with Curie temperatures well below room temperature [2,6], the origin of ferromagnetism (in some cases, even at and above room temperature) in (ii) remains still controversial [9,10]. In type (ii) DMSs, it has been argued that the presence of ferromagnetic clusters of metallic Fe, Co, or Ni could be a plausible reason for the observed ferromagnetic properties when using ferromagnetic dopants [11]. However, even when the doping element is not ferromagnetic (e.g., Cu), ferromagnetic behavior has been also reported in these materials, albeit unrelated to the 3d moment [12]. Additionally, it has been claimed that undoped oxide semiconductors may also exhibit ferromagnetic properties [13]. Some studies point to structural defects (such as oxygen vacancies) as the source of the observed ferromagnetism [7,14,15], whereas other investigations link the magnetic response to either ferromagnetic impurities or instrumental artifacts since the involved magnetic signal is usually close to the sensitivity limit of state-of-the-art magnetometry setups [9,10].

Most oxide DMSs reported so far have been prepared as continuous thin films, bulk materials, or coarse polycrystalline powders. Although there are a few studies on the growth of oxide DMS nanoparticles [16] and nanowires [17], the synthesis of three-dimensional (3D) mesoporous oxide DMS structures by hard-templating has been just recently reported [18,19]. Engineering 3D magnetic semiconductor architectures with ordered arrangements (i.e., controlled size, shape, and orientation of the pores and pore walls) is highly desirable since this allows for the precise tuning of the physicochemical properties [20]. Porous oxide DMS frameworks are expected to exhibit coupled electronic and magnetic properties, quantum confinement effects, a high internal surface area for absorption purposes, and novel synergetic properties arising from the possibility of filling the internal pores with secondary host materials. 

Among the wide range of oxide semiconductors, we focus our attention on tin dioxide (SnO_2_), n–type semiconductor (band gap E_g_ = 3.6 eV at 300 K) with tetragonal rutile structure [21,22] that has been one of the most investigated materials due to its fascinating optical and electrical properties [23,24]. In particular, SnO_2_ has been largely used in solid-state gas devices owing to its mechanical hardness, electrical resistivity, and chemical inertness (e.g., as CO detector) [25]. However, one of the key issues limiting its wide gas-sensing applications is its lower selectivity and durability. Thus, much effort has been devoted to the enhancement of its gas-sensing performance by suitably doping it with noble metals, semi-metals or halogens [26,27,28], or by increasing its surface-to-volume ratio. Typically, ordered mesoporous SnO_2_ particles have been obtained as a negative replica of SBA–15 [29] and KIT–6 [30] mesoporous silica templates and chloride precursors (e.g., SnCl_2_·2H_2_O). However, the synthesis of mesoporous SnO_2_ from MCM–41 or cage–like SBA–16 silica templates has also been reported [31,32]. Nowadays, although mesoporous SnO_2_ has been widely investigated for its potential application in many different fields [33,34,35,36,37,38], magnetism and spintronics studies for this material are overlooked.

In this article, we report on the preparation of mesoporous Cu-doped SnO_2_ DMS powders by nanocasting and we investigate the origin of the observed magnetic behavior at room and low temperatures, with the use of magnetometry, and the element selective method of X-ray magnetic circular dichroism (XMCD).

## 2. Experimental Details

### 2.1. Materials 

HCl (Hydrochloric acid, 37 wt %), 1-butanol (99.9%), TEOS (Tetraethylorthosilicate, 99.0%), SnCl_2_·2H_2_O (stannous chloride dihydrate, 99.99%), CuCl_2_·2H_2_O (copper chloride dihydrate, 99.0%), Pluronic P-123 (HO(CH_2_CH_2_O)_20_(CH_2_CH(CH_3_)O)_70_(CH_2_CH_2_O)_20_H) block copolymer, and absolute ethanol (99.8%) were purchased from Sigma-Aldrich (Saint Louis, MO, USA). All of the reagents were used as-received without further purification. Deionized water was obtained through an EMD Millipore Simplicity™ Water Purification System (Millipore S.A.S., Molsheim 67120, France).

### 2.2. Synthesis of Mesoporous Cu-Doped SnO_2_ Powders

Mesoporous KIT–6 silica (synthesized in our lab) [18] was used as a hard template. SnCl_2_ was chosen as the precursor of SnO_2_ and CuCl_2_ as the doping agent. First, KIT–6 silica (0.4 g) was mixed with SnCl_2_·2H_2_O (0.6 g) and different amounts of CuCl_2_·2H_2_O (molar ratios of Cu versus Sn reagents were 0:100, 5:95, 15:85, and 20:80). The mixture was finely ground in an agate mortar and pestle, and then placed in a ceramic crucible and put into a vacuum furnace (pressure <10^−4^ mbar) to promote the infiltration of the precursors in the silica host. The heating temperature and time were set at 85 °C and 24 h, respectively [32,39]. Afterwards, the crucible, containing the sample, was transferred to a tubular furnace to convert the tin and copper precursors into the oxides. The calcination conditions were set at 700 °C under air atmosphere for 2 h, with a heating rate 2 °C/min. When the heating process was finished, 1 M NaOH aqueous solution was prepared for etching away the KIT–6 silica template at 70 °C with mild stirring. Finally, the resulting powders were cleaned with deionized water and absolute ethanol and dried in an oven.

### 2.3. Characterization

Scanning electron microscopy (SEM) and transmission electron microscopy (TEM) were employed to analyse the morphology and microstructure of the powders. SEM observations were carried out on a Zeiss Merlin microscope (Jena, Germany) that was equipped with an energy dispersive X-ray detector (EDX). TEM observations were performed on a JEOL-JEM 2011 (JEOL USA, Inc., Peabody, MA, USA) operated at 200 kV. High-resolution electron microscopy images were obtained on Tecnai F20 microscope (HR-STEM) equipped with selected area electron diffraction (SAED). θ/2θ X-ray diffraction (XRD) patterns were obtained on a PANalytical X’Pert powder diffractometer (PANalytical B.V., Almelo, The Netherlands) that was equipped with Cu K_α_ radiation (λ = 0.154 nm). Rietveld refinements of the full XRD patterns were performed using the “Material Analysis using Diffraction” (MAUD) software [40,41] to extract the values of crystallite size and lattice parameters as a function of the Cu doping. X-ray photoelectron spectroscopy (XPS) analyses were carried out in a spectrometer PHI 5500 Multitechnique System(Perkin–Elmer, Waltham, MA, USA) that was equipped with a monochromatic X-ray source (K_α_ Al line of 1486.6 eV energy and 350 W), which was placed perpendicular to the analyser axis and calibrated using the 3d_5/2_ line of Ag, with a full width at half maximum (FWHM) of 0.8 eV. All of the measurements were done under ultra-high vacuum (UHV), with pressure between 5 × 10^−9^ and 2 × 10^−8^ Torr. The analysed area was a circular spot of 0.8 mm in diameter for each sample. Peaks were corrected to the position of adventitious C 1s signal (284.5 eV) [42,43]. Experimental core-level spectra were fitted using Gaussian curves. The magnetic properties of the samples were investigated by means of superconducting quantum interference device (SQUID) magnetometry at room (300 K) and cryogenic (5 K) temperatures (Quantum Design MPMS XL-7T setup). Further magnetic characterization was carried out by X-ray magnetic circular dichroism (XMCD), which records the difference in core-level absorption spectra between right-handed (μ^+^) and left-handed (μ^−^) circularly polarized X-rays. Specifically, Cu L_3,2_ edge X-ray absorption spectra (XAS), measured in total electron yield (TEY) mode for right (μ^+^) and left (μ^−^) circularly polarized light, were taken at the UE46_PGM1 beamline (High-Field Diffractometer station of the synchrotron radiation source BESSY II, Helmholtz-Zentrum Berlin). The XMCD experiments were performed at room (300 K) and cryogenic (5 K) temperatures, under the applied magnetic fields of 5 and −5 kOe. Since the powder samples arerather insulating, they revealed temperature and specimen dependent charging effects, even though they were placed onto conductive Au/Si substrates. These refer to the measured intensity, and in particular, the shape of the background [44]. However, since both of the light polarizations are affected in the same way, the XMCD obtained by TEY allows for a meaningful comparison. The presented absorption spectra for both right (μ^+^) and left (μ^−^) circularly polarized light are the average of two spectra. 

Both the SQUID and XMCD low temperature measurements were carried out by cooling from room temperature down to 5 K in an applied magnetic field of 5 kOe with the aim to generate a preferential direction stemming from uncompensated spins in the antiferromagnetic order.

## 3. Results and Discussion

### 3.1. Morphological and Structural Characterization

**Figure 1:**
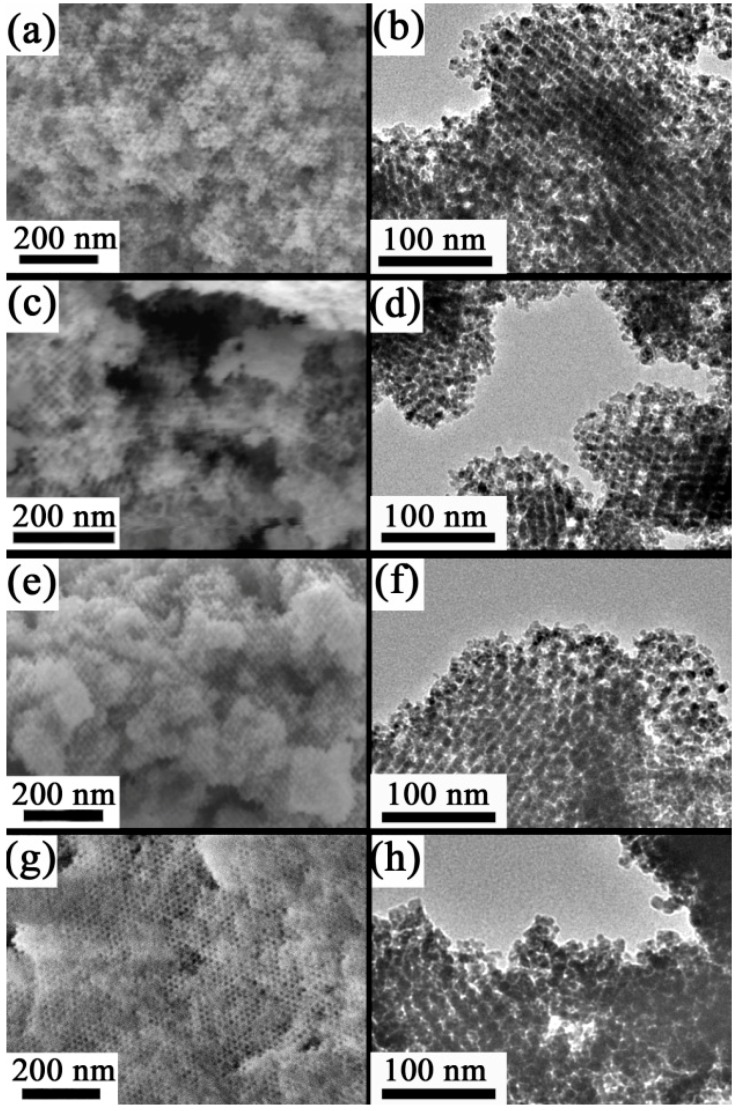
Morphology of Cu-doped SnO_2_ powders after KIT–6 silica removal. Panels (**a**,**c**,**e**,**g**) are the scanning electron microscopy (SEM) images of the powders obtained from different [Cu(II)]:[Sn(II)] molar ratios (0:100, 5:95, 15:85, and 20:80, respectively). Panels (**b**,**d**,**f**,**h**) are the corresponding transmission electron microscopy (TEM) images.

The morphology of the Cu-doped SnO_2_ powders was examined by scanning/transmission electron microscopies (SEM/TEM). Figure 1a,c,e,g shows the SEM images of samples synthesized from 0:100, 5:95, 15:85, and 20:80 [Cu(II)]:[Sn(II)] molar ratios, respectively. In turn, Figure 1b,d,f,h displays their corresponding TEM images. In all of the cases, a highly ordered mesoporous arrangement of pores is preserved after the KIT–6 silica template removal. It is noteworthy that Cu-loading does not significantly affect the mesoporous structure of the SnO_2_ replica. The copper amounts in at.%, determined by energy dispersive X-ray analysis (EDX), for the different samples, are listed in Table 1. As expected, the Cu contents become larger (from 0 to 7 at.%), when the [Cu(II)]:[Sn(II)] molar ratio increases from 0:100 to 20:80. 

**Figure 2:**
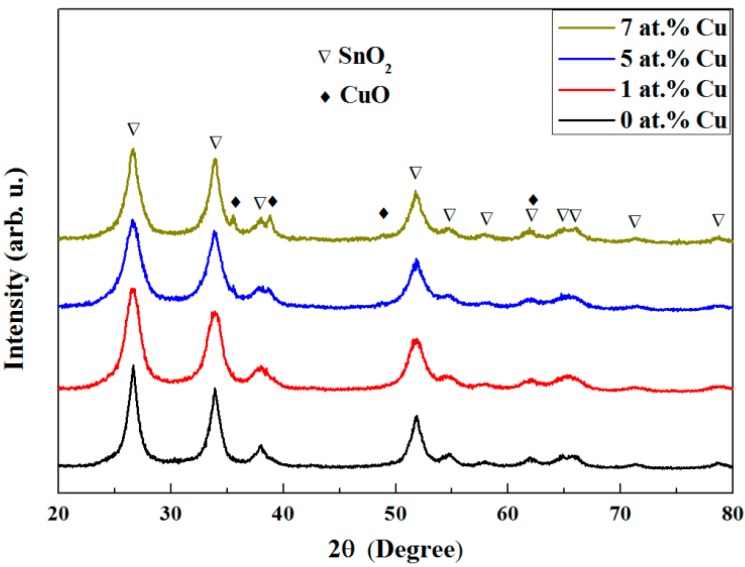
θ/2θ XRD patterns of the mesoporous SnO_2_ powders doped with 0 (undoped), 1, 5 and 7 at.% Cu.

To further investigate the microstructure and the crystallographic phases of the samples, X-ray diffraction (XRD) analyses were performed. The main XRD peaks correspond to the SnO_2_ rutile-type tetragonal phase (JCPDS No. 88-0287). Traces of CuO (JCPDS No. 01-1117) might be envisaged in the 1 at.% Cu SnO_2_ sample and these XRD peaks become more defined and sharper with further Cu doping, suggesting a formation of a more stoichiometric CuO (Figure 2). Information on the crystallite size (average coherently diffracting domain size) and lattice parameters (a = b and c) for SnO_2_ was obtained by Rietveld refinement of the θ/2θ XRD patterns (Table 1). The SnO_2_ crystallite size of the samples evidences their nanoscale character, slightly decreasing after Cu doping, in agreement with the formation of a secondary CuO phase. Even though no significant differences are observed in the lattice cell parameters, Cu incorporation into the mesoporous SnO_2_ lattice cannot be fully ruled out since the ionic radii of Cu^2+^ and Sn^4+^ are rather similar (73 and 69 pm, respectively). To further shed light into the structure, the samples were investigated by TEM.

**Figure 3:**
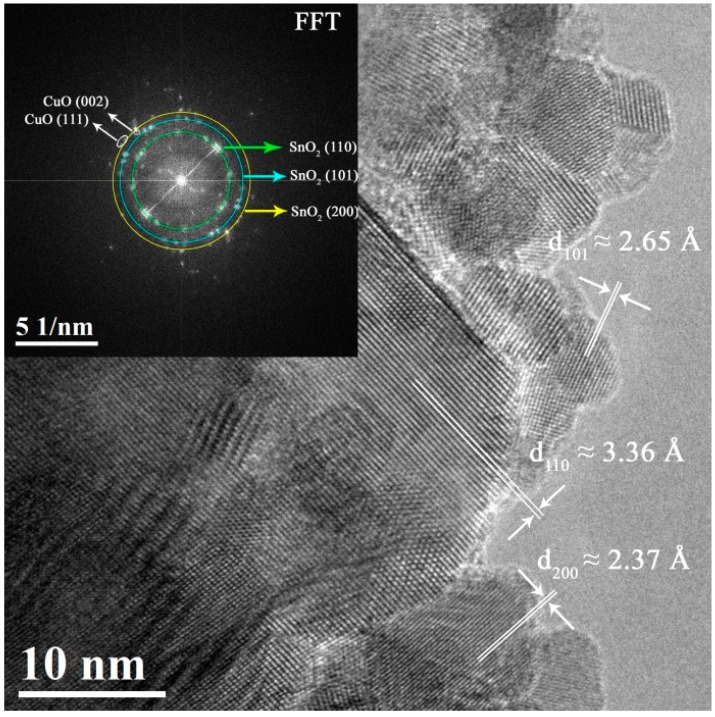
TEM image and corresponding fast Fourier transform (FFT) of the 7 at.% Cu-doped SnO_2_ powders.

Figure 3 shows a TEM image and its corresponding fast Fourier transform (FFT) for the mesoporous SnO_2_ sample with the highest Cu-content (i.e., 7 at.% Cu). Analogous images corresponding to the samples with 1 and 5 at.% Cu are shown in the Supporting Information (Figures S1 and S2, respectively). The sample contains highly crystalline SnO_2_ nanoparticles of ca. 10 nm, with typical interplanar distances of the tetragonal phase (d_110_ = 3.36 Å, d_101_ = 2.65 Å and d_200_ = 2.37 Å). Additionally, the FFT (inset of Figure 3) reveals, besides SnO_2_, spots arising from CuO planes [(i.e., (100) and (002)), in concordance with the XRD results.

**Figure 4:**
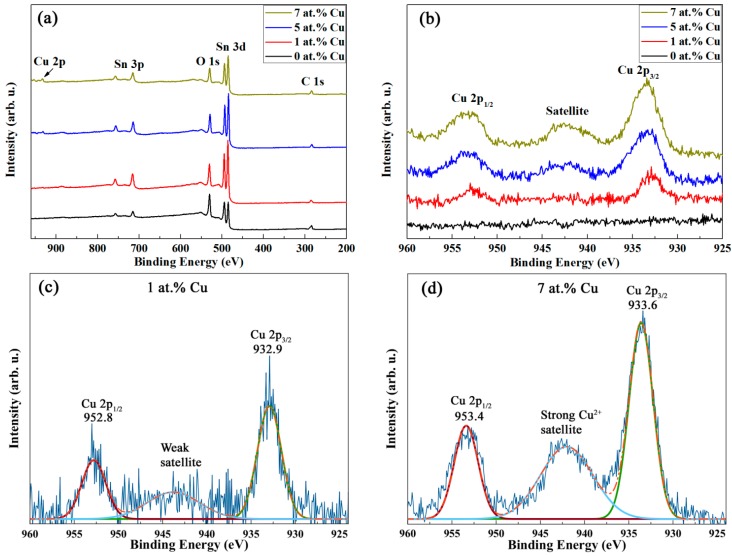
(**a**) General X-ray photoelectron spectroscopy(XPS) survey spectra of undoped and Cu-doped SnO_2_ mesoporous powders. (**b**) High resolution XPS spectra of the Cu 2p level. (**c**,**d**) are the Cu 2p deconvolutions corresponding to the 1 and 7 at.% Cu SnO_2_ samples, respectively.

X-ray photoelectron spectroscopy (XPS) was used to investigate the valence states of Cu. Because ion bombardment may cause the reduction of CuO (i.e., Cu^2+^) to Cu^1+^ [45], XPS was carried out without any pre-sputtering. The general XPS survey spectra of the various samples are shown in Figure 4a. Cu 2p peaks emerge and gradually increase with Cu doping. As can be seen in the high resolution core-level spectra of Cu 2p (Figure 4b), Cu 2p_3/2_, and Cu 2p_1/2_ peaks slightly shift towards higher energies with the increase of doping amount. In addition, a satellite peak from Cu^2+^ centred at around 942 eV appears, and its relative intensity is also enhanced with the increase of Cu content. Figure 4c,d show the high resolution spectra of Cu 2p together with the peak deconvolution of the 1 and 7 at.% Cu samples, respectively. The sample with 7 at.% Cu exhibits a Cu 2p_3/2_ binding energy of around 933.6 eV, which is consistent with CuO [46,47]. Conversely, for the sample with 1 at.% Cu, the binding energy of Cu 2p_3/2_ is 932.9 eV, a slightly lower value, which falls in an energy range characteristic of mixed Cu valence states (i.e., Cu^1+^ and Cu^2+^) [48,49] and, thus, representing a mixture of Cu_2_O and CuO. Furthermore, partial non-stoichiometry is likely to be caused by oxygen vacancies associated with the synthesis method [50,51], as also evidenced in mesoporous transition metal-doped In_2_O_3_ and Ni-doped SnO_2_ [18,19].

### 3.2. Room and Low Temperature Magnetic Properties

**Figure 5:**
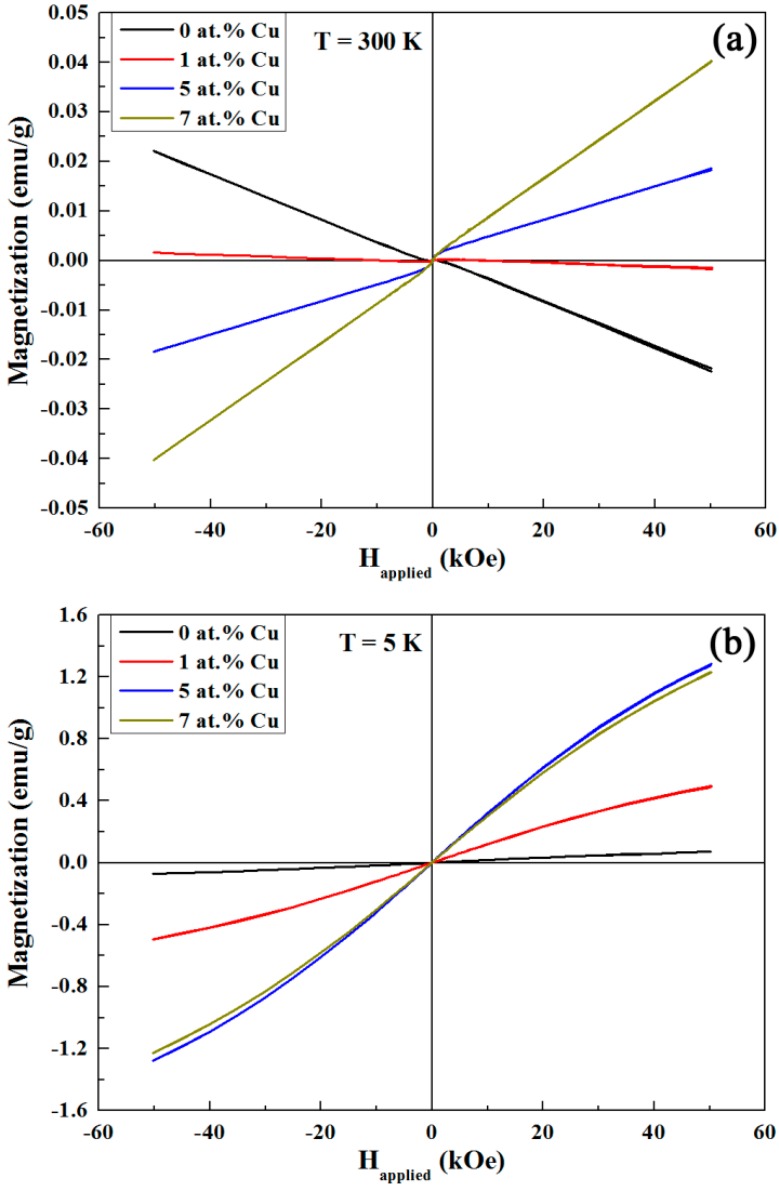
(**a**) Room temperature (300 K) and (**b**) 5 K SQUID measurements of the ordered mesoporous Cu-doped SnO_2_ powders containing 0 (undoped), 1, 5, and 7 at.% Cu. The low temperature hysteresis loops were taken upon cooling from 300 K down to 5 K in an applied magnetic field of 50 kOe.

Figure 5a shows the room temperature (RT) magnetization (M) vs. applied magnetic field (H_applied_) raw curves recorded by SQUID magnetometry of the mesoporous SnO_2_ powders containing 0 (undoped), 1, 5, and 7 at.% Cu. In contrast to the samples with 5 and 7 at.% Cu, the magnetization at high fields of the undoped and Cu-doped (1 at.%) SnO_2_ samples decreases with the applied magnetic field, confirming the diamagnetic character of the SnO_2_. However, the slope M/H_applied_ of the sample doped with 1 at.% Cu is significantly larger than that of the undoped sample, evidencing a substantial Cu magnetic moment that reduces the total diamagnetic response when compared to the pure SnO_2_. This would be expected from paramagnetic CuO [12,52], as indicated by traces of CuO XRD peaks in Figure 2 and a satellite peak corresponding to the presence of Cu^2+^ in the XPS measurements (Figure 4c). In the samples with higher doping levels of 5 and 7 at.% Cu, the diamagnetism of SnO_2_ is even overcome resulting in an effective paramagnetic response (positive M/H_applied_ slope at high fields) ascribed to a larger amount of paramagnetic phase (i.e., CuO). This is in full agreement with the XRD (Figure 2) and XPS (Figure 4d) results, which show clear CuO XRD peaks and a well-defined Cu^2+^ peak, respectively, at the higher doping levels.

All of the samples exhibit a weak hysteretic behavior at RT superimposed to the diamagnetic or paramagnetic backgrounds, (i.e., mild RT ferromagnetism) whose origin remains rather intricate since no ferromagnetic phases are apparently involved. Some studies point to structural defects (such as oxygen vacancies) as the source of the observed ferromagnetism [8,9], whereas other investigations link it to either ferromagnetic contamination, arising from sample handling and/or the impurity of precursors, or instrumental artifacts [9,10]. Inductively coupled plasma mass spectrometry (ICP-MS) measurements evidence the existence of Fe traces (of the order of 200–400 ppm) in all the samples, which could explain the weak RT ferromagnetic behavior. Namely, for instance, after subtracting the linear background of the measurement corresponding to the powders doped with 7 at.% Cu, a saturation magnetization (M_S_) of around 9.5 × 10^−4^ emu/g is obtained. Upon the assumption that ferromagnetism may solely arise from Fe contamination (M_S_ of 217.2 emu/g at 298 K) [53], just 33 ppm of Fe would be sufficient to obtain that M_S_. However, the lack of information on the Fe species (metallic versus oxide character) and morphology does not allow us to be completely conclusive on this issue, hence leaving the origin of the weak RT ferromagnetism open and possibly related to oxygen vacancies.

Figure 5b shows the raw SQUID measurements of the mesoporous SnO_2_ powders containing 0 (undoped), 1, 5, and 7 at.% Cu carried out 5 K after field cooling from RT in an applied magnetic field of 50 kOe. Remarkably, the magnetization at high fields is significantly larger than that at RT (in particular, for the doped samples which contain CuO). For example, upon subtraction of the linear background of the measurement at high applied magnetic fields, the powders doped with 7 at.% Cu exhibit a saturation magnetization (M_S_) of 0.32 emu/g. This value is more than 300 times larger than that at RT (9.5 × 10^−4^ emu/g). For metallic bulk iron, the saturation magnetization at 0 K is only 1.02 times larger than that of RT [53]. Therefore, this suggests the presence of another source of magnetic moment rather than iron at a low temperature, and, the CuO present in the doped samples is a clear candidate as it shows low temperature antiferromagnetic order. Incommensurate helix-like antiferromagnetism is observed below 230 K (Néel temperature T_N2_) down to 213 K (Néel temperature T_N1_). Below T_N1_, CuO shows commensurate antiferromagnetic order [54]. Even though no net magnetization is expected in CuO, low-dimensional (i.e., nanoscale) forms of CuO might give rise to a net magnetic moment due to size effects [52,55]. Among them, the presence of uncompensated spins at the surface ascribed to low coordination of surface sites and shape-mediated spin canting, as it happens with other antiferromagnets in nanoscale form (BiFeO_3_ [56] or NiO [57]), can result in ferromagnetic-like behavior. When comparing the magnetization at high fields for the Cu-doped samples, it is clear that the signal is much higher for the 5 and 7 at.% Cu powders than for the 1 at.% Cu sample, evidencing that the contribution of uncompensated spins and spin canting is larger and similar for the 5 and 7 at.% Cu samples. The magnetization of these samples scales with the amount of Cu at room temperature, whereas, at a low temperature, the sample with 5 at.% Cu shows a slightly larger saturation magnetization than that of the 7 at.% Cu. This rules out a spin-1/2 paramagnetic behavior [58] and, thus, further confirms the presence of magnetic order at low temperature. 

A common feature of these nanoscale antiferromagnets is spin frustration, which usually results in vertical shifts (in particular upon field cooling from a temperature above T_N_) [59,60]. Actually, a little vertical shift of around 3 × 10^−4^ emu/g towards positive M is observed in the powders with 7 at.% Cu, corroborating the size effects in the formed CuO. A weaker vertical shift is also observed in the samples with 1 and 5 at.% Cu (being smaller in the sample with lower Cu content), whereas the undoped SnO_2_ powders exhibit no vertical asymmetry.

To further investigate the origin of the magnetic properties, an element-specific synchrotron technique was employed. Namely, XMCD at the Cu L_2,3_ edge was performed at the UE46_PGM1 beamline (High-Field Diffractometer station of the synchrotron radiation source BESSY II). Since the powders containing 5 and 7 at.% Cu show a similar magnetic behavior, the samples with 1 and 7 at.% Cu were selected for XMCD measurements.

**Figure 6:**
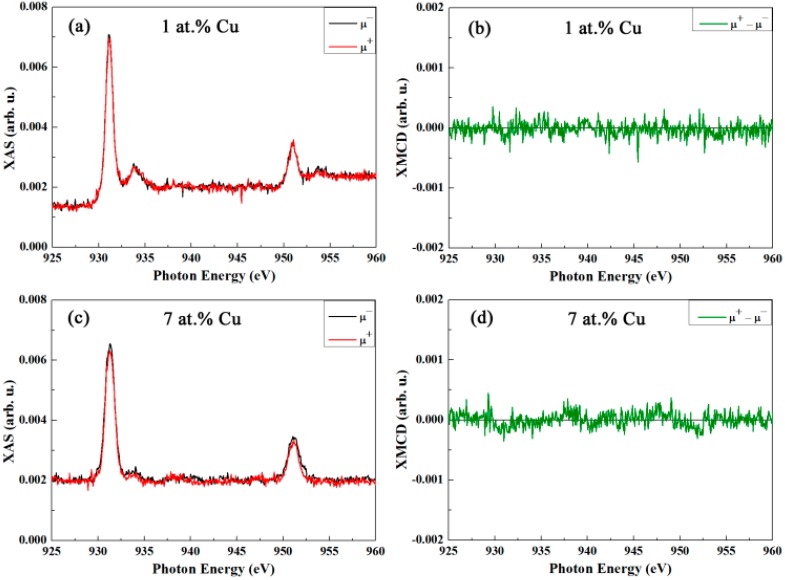
(**a**,**c**) Cu L_3,2_ edge X-ray absorption spectroscopy(XAS) spectra, measured in total electron yield mode for right (μ^+^) and left (μ^−^) circularly polarized light, recorded at room temperature in an applied magnetic field of 50 kOe for the SnO_2_ powders doped with (**a**) 1 at.% Cu and (**c**) 7 at.% Cu, respectively. (**b**,**d**) are the corresponding X-ray magnetic circular dichroism(XMCD) signals (i.e., difference between right and left circularly polarized light) for the SnO_2_ powders doped with 1 and 7 at.% Cu, respectively.

Figure 6a,c shows the room temperature Cu L_3,2_ edge XAS spectra for right (μ^+^) and left (μ^−^) circularly polarized light corresponding to the samples with 1 and 7 at.% Cu, respectively, obtained under an applied magnetic field of 50 kOe. Figure 6b,d represent the XMCD signal, taken as the difference (in arbitrary units) between the right and left circularly polarized spectra presented in Figure 6a,c respectively. The XAS spectra of the samples doped with 1 and 7 at.% of Cu (Figure 6a,c respectively) are consistent with a predominant CuO phase [61,62,63,64]. However, traces of Cu in 1+ valence (peak at around 934.5 eV) are present in the sample doped with 1 at.% Cu [61,62,63,64]. This is in agreement with the XRD and XPS characterization where only clear crystalline peaks of CuO and a well-defined Cu^2+^ signal are observed in the samples with higher Cu contents. For both of the samples, the absorption intensity is independent of the light polarization (i.e., no asymmetry in the intensity between the right (μ^+^) and left (μ^−^) circularly polarized X-ray absorption spectra), indicating no dichroism in copper and, thus, no ferromagnetic behavior in agreement with the paramagnetic character of CuO at room temperature.

**Figure 7:**
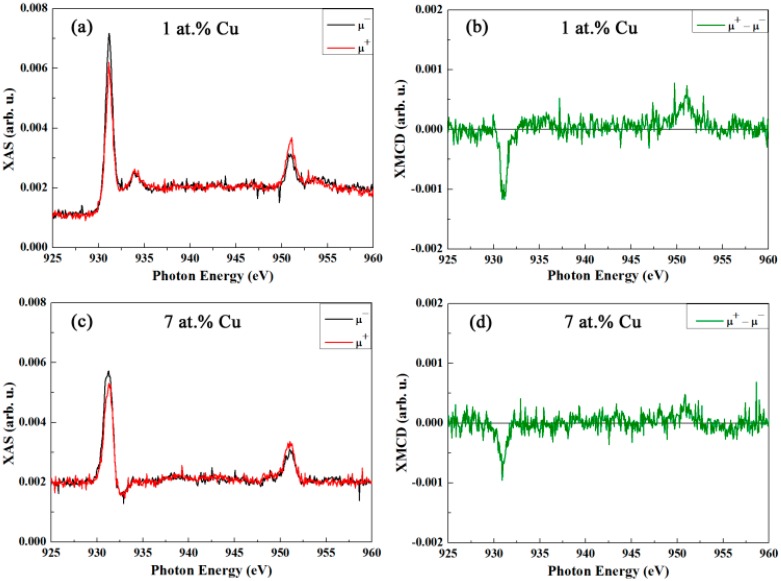
(**a**,**c**) Cu L_3,2_ edge XAS spectra, measured in total electron yield mode for right (μ^+^) and left (μ^−^) circularly polarized light, recorded at 5 K (after cooling in 50 kOe) under an applied magnetic field of 50 kOe for the SnO_2_ powders doped with (**a**) 1 at.% Cu and (**c**) 7 at.% Cu, respectively. (**b**) and (**d**) are the corresponding XMCD signals at 5 K (i.e., difference between right and left circularly polarized light) for the SnO_2_ powders doped with 1 and 7 at.% Cu, respectively.

Conversely, as can be seen in Figure 7, there is a pronounced intensity asymmetry between the right (μ^+^) and left (μ^−^) circularly polarized X-ray absorption spectra for both samples at 5 K, evidencing a significant dichroism in Cu and, consequently, a magnetic moment at the Cu site. The temperature of the measurement (i.e., 5 K) is well below the bulk Néel temperatures of the CuO, therefore, even an ordered Cu moment may be anticipated. Due to finite size effects, uncompensated Cu moments as well as spin canting may lead to a net magnetization. 

**Figure 8:**
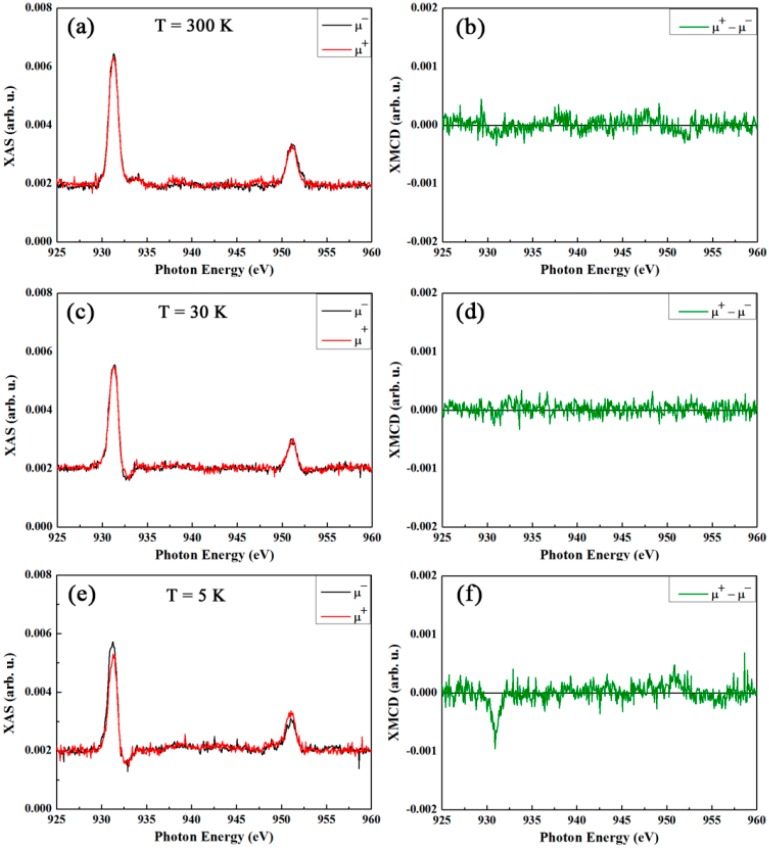
Cu L_3,2_ edge XAS spectra of the powders doped with 7 at.% Cu, measured in total electron yield mode for right (μ^+^) and left (μ^−^) circularly polarized light, recorded at 300 (**a**), 30 (**c**) and 5 K (**e**) under an applied magnetic field of 50 kOe. The cooling was done in 50 kOe. (**b**,**d**,**f**) are the corresponding XMCD signals (i.e., difference between right and left circularly polarized light).

The presence of ordered Cu moments in contrast to paramagnetic behavior is further corroborated by the evolution of the XMCD with temperature, as shown in Figure 8, for the sample doped with 7 at.% Cu. Remarkably, the XMCD signal (i.e., Cu dichroism) has already vanished at 30 K, ruling out a paramagnetic behavior, and rather indicating a connection with Cu ordering. This, in fact, then indicates a strongly reduced Néel temperature, or, when physical confinement plays a role, a blocking temperature. Hence, the formed CuO is highly affected by size effects in agreement with the structural characterization. These XMCD results suggest an ordered, ferromagnetic-like phase of Cu moments, with a finite magnetization. It is to be noted, however, that any possible hysteretic behavior at low temperature escaped unambiguous detection within the experimental limitations.

Further support for size effects on this low-temperature ferromagnetic-like response in CuO may be obtained from a quantification of the XMCD, as presented in Table 2, which shows the relative signals of the SnO_2_ powders doped with 1 and 7 at.% Cu measured at 5 K under 50 and −50 kOe. The relative magnitude of the XMCD was quantified as explained in the Electronic Supplementary Information (Figure S3 and Table S1). As expected, the XMCD signal for the sample with 7 at.% Cu is larger than for the one with 1 at.% Cu (Table 2). This is ascribed to the larger amount CuO, which is probably also more stoichiometric. Note that traces of Cu^1+^ are only observable in the XAS spectra of the sample containing 1 at.% of Cu.

As aforementioned, a common feature of nanoscale antiferromagnets is to show hysteresis loops with vertical shifts which ultimately stem from size effects. As it happens with the SQUID characterization, the XMCD analysis also reveals this phenomenon. Namely, the XMCD signal at +50 kOe is significantly larger than at −50 kOe (Table 2), confirming spin frustration upon reversal due to size effects. This is more pronounced in the sample with higher Cu content, suggesting that, at higher doping concentrations, a more stoichiometric CuO phase with a better defined magnetic anisotropy is formed [65].

## 4. Conclusions

Ordered mesoporous Cu-doped SnO_2_ powders have been satisfactorily prepared by hard-templating from KIT-6 silica. While Fe contamination or the presence of oxygen vacancies could be plausible explanations for the room temperature ferromagnetism, the observed low temperature ferromagnetic-like behavior arises from nanoscale CuO, where finite size effects yield a net magnetization, as evidenced by XMCD at the Cu L_3,2_ resonances. This ferromagnetic-like behavior is primarily ascribed to both uncompensated spins and shape-mediated spin canting. The reduced blocking temperature, which resides between 30 and 5 K, and traces of vertical shifts in the hysteresis loops confirm the size effects in CuO. The possibility to induce magnetic order in DMSs by introducing 3d metals in nanoscale dimension using mesoporous powders offers new prospects in the field of spintronics as the amount of surface area-to-volume ratio is highly increased, rendering potential for novel applications that could be based on magnetic surface effects.

## Figures and Tables

**Table 1:** Atomic percentages of Cu assessed by energy dispersive X-ray analysis (EDX), crystallite size and lattice cell parameters of the SnO_2_ phase (determined by Rietveld refinement of the X-ray diffraction (XRD) patterns) of the samples synthesized from [Cu(II)]:[Sn(II)] molar ratios of 0:100, 5:95, 15:85, and 20:80.

[Cu(II)]:[Sn(II)]	Cu Content Determined by EDX (at.%)	Crystallite Size SnO_2_ Phase (nm) (±1)	a (Å) SnO_2_ Phase (±1 × 10^−3^)	c (Å) SnO_2_ Phase (±1 × 10^−3^)
0:100	0	9	4.737	3.187
5:95	1	7	4.739	3.189
15:85	5	6	4.739	3.189
20:80	7	7	4.737	3.186

**Table 2:** Relative XMCD signals and corresponding errors of the SnO_2_ powders doped with 1 and 7 at.% Cu measured at 5 K under 50 and −50 kOe. See supplementary information for details on the calculation of the XMCD signal and its error.

	5 K			
	1 at.% Cu		7 at.% Cu	
	50 kOe	−50 kOe	50 kOe	−50 kOe
**XMCD ± δXMCD**	36 ± 2%	29 ± 2%	39 ± 3%	32 ± 4%

## Supplementary Materials

The following are available online at www.mdpi.com/2079-4991/7/11/348/s1, Figure S1: TEM image and corresponding FFT of the 1 at.% Cu-doped SnO_2_ powders; Figure S2: TEM image and corresponding FFT of the 5 at.% Cu-doped SnO_2_ powders; Figure S3: (a) Cu L_3,2_ edge X-ray absorption spectroscopy (XAS) spectra, measured in total electron yield mode for right (μ^+^) and left (μ^−^) circularly polarized light, recorded at 5 K (after cooling in 50 kOe) under an applied magnetic field of 50 kOe for the SnO_2_ powders doped with 7 at.% Cu; (b) is the corresponding relative XMCD signal (i.e., difference between right and left circularly polarized light). The parameters to quantify the relative XMCD signal are also presented.; Table S1: y_1_ and y_2_ values for the XMCD signal quantification corresponding to the samples with 1 and 7 at.% Cu measured at 5 K applying a magnetic field equal to 50 and −50 kOe.
