# Supplementary material for: Unraveling the Origin of Magnetism in Mesoporous Cu-Doped SnO2 Magnetic Semiconductors
Source: Nanomaterials (Basel). 2017 Oct 25;7(11):348. doi: 10.3390/nano7110348 (PMC5707565; doi:10.3390/nano7110348)
Supplement: Supplementary file 1 [file nanomaterials-07-00348-s001.pdf]

Unraveling the origin of magnetism in mesoporous Cu-doped SnO_2 magnetic semiconductors

J. Fan[1], E. Menéndez[1,*], M. Guerrero[1], A. Quintana[1], E. Weschke[2], E. Pellicer[1], J. Sort[1,3]

1. TEM of other samples

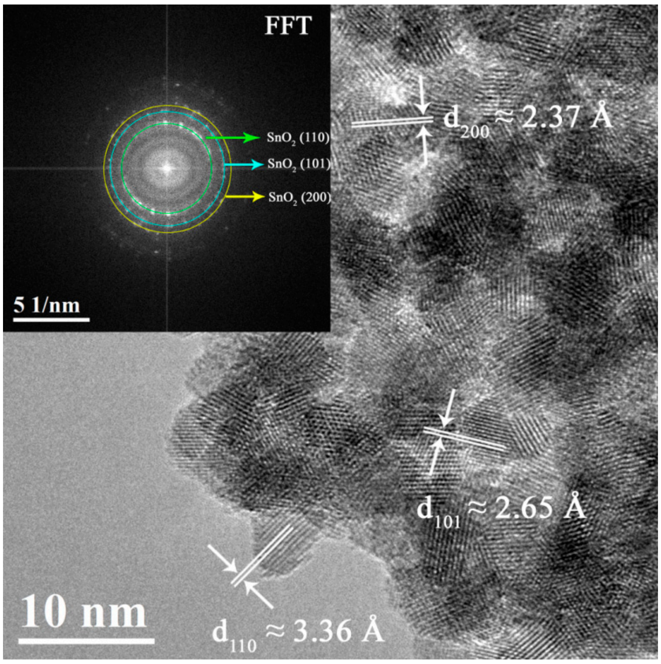

Figure S1. TEM image and corresponding FFT of the 1 at.% Cu-doped SnO_2 powders.

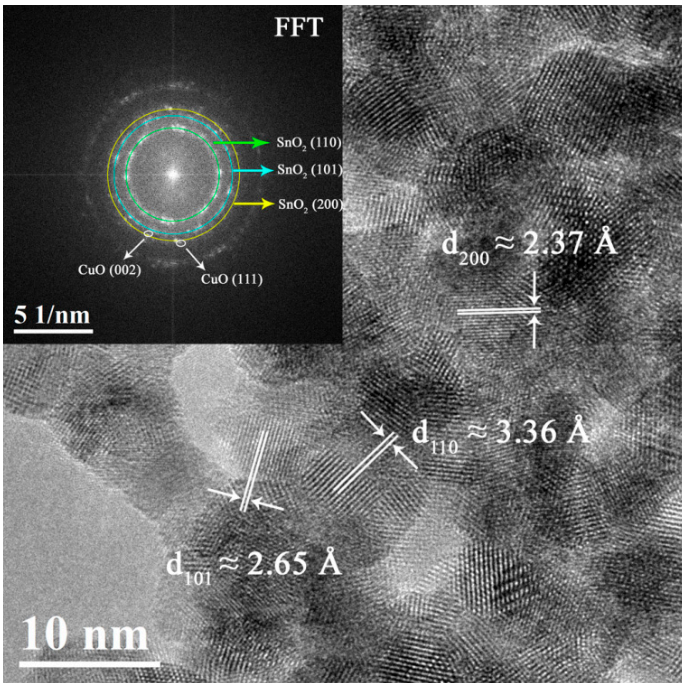

Figure S2. TEM image and corresponding FFT of the 5 at.% Cu-doped SnO_2 powders.

2. Further details on the XMCD analysis

The presented absorption spectra for both right (μ^+) and left (μ^-) circularly polarized light are the average of two spectra. As can be seen in Figure S3, the relative XMCD signal is defined as the amplitude (y_1) between the valley and the peak of the difference between the right and left circularly polarized spectra (i.e., $\mu^+ - \mu^-$) divided by the average of the Cu L_3 absorption peaks y_2 (i.e., $y_2 = \left(\frac{y_2^- + y_2^+}{2}\right)$). Thus,

$$\mathrm{XMCD}(\%) = \left(\frac{y_1}{y_2}\right) \cdot 100\ \%$$

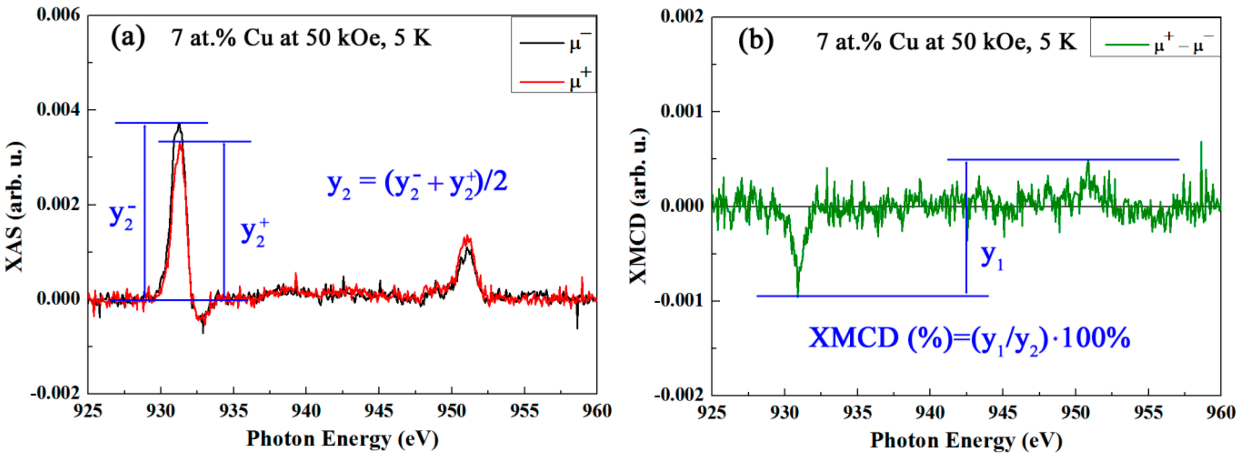

Figure S3. (**a**) Cu $L_{3,2}$ edge X-ray absorption spectroscopy (XAS) spectra, measured in total electron yield mode for right (μ^+) and left (μ^-) circularly polarized light, recorded at 5 K (after cooling in 50 kOe) under an applied magnetic field of 50 kOe for the SnO_2 powders doped with 7 at.% Cu. (**b**) is the corresponding relative XMCD signal (i.e., difference between right and left circularly polarized light). The parameters to quantify the relative XMCD signal are also presented.

The error in the relative XMCD signal quantification is propagated assuming dependent errors among the y_1 and y_2 variables. Taking into account that:

$$\mathrm{XMCD}(\%) = \left(\frac{y_1}{y_2}\right) \cdot 100\ \%, \text{ then:}$$

$$\delta[\mathrm{XMCD}(\%)] = \left(\left|\frac{1}{y_2}\right| \delta y_1 + y_1 \left|-\frac{1}{{y_2}^2}\right| \delta y_2\right) \cdot 100\ \%$$

$$\delta y_2 = \frac{1}{2}(\delta y_2^+ + \delta y_1^-)$$

δy_2^+ and δy_2^- are taken as half of the amplitude of the background signal which, for both samples, is around 5×10^{-5}. Hence, $\delta y_2 = 5\times10^{-5}$. δy_1 is twice δy_2 because it involves the difference of two spectra (i.e., $\mu^+ - \mu^-$). Thus, $\delta y_1 = 1\times10^{-4}$.

Table S1. y_1 and y_2 values for the XMCD signal quantification corresponding to the samples with 1 and 7 at.% Cu measured at 5 K applying a magnetic field equal to 50 and −50 kOe.

	**5 K**			
	**1 at.% Cu**		**7 at.% Cu**	
	**50 kOe**	**−50 kOe**	**50 kOe**	**−50 kOe**
**y_1**	2.0×10^{-3}	1.5×10^{-3}	1.4×10^{-3}	1.0×10^{-3}
**y_2**	5.56×10^{-3}	5.19×10^{-3}	3.55×10^{-3}	3.17×10^{-3}
